# Modeling the Burden of Cardiovascular Diseases in Iran from 2005 to 2025: The Impact of Demographic Changes

**Published:** 2017-04

**Authors:** Masoumeh SADEGHI, Ali Akbar HAGHDOOST, Abbas BAHRAMPOUR, Mohsen DEHGHANI

**Affiliations:** 1. Modeling in Health Research Center, Institute for Futures Studies in Health, Kerman University of Medical Sciences, Kerman, Iran; 2. Health Services Management Research Center, Institute for Futures Studies in Health, Department of Biostatistics and Epidemiology, Kerman University of Medical Sciences, Kerman, Iran; 3. Social Determinants of Health Research Center, Institute for Futures Studies in Health, Kerman University of Medical Sciences, Kerman, Iran; 4. Dept. of Biostatistics and Epidemiology, Kerman University of Medical Sciences, Kerman, Iran; 5. Center for Health Related Social and Behavioral Sciences Research, Shahroud University of Medical Sciences, Shahroud, Iran

**Keywords:** Cardiovascular diseases, Iran, Demographic changes, DALY, Modelling

## Abstract

**Background:**

Estimating the burden of non-communicable diseases particularly cardiovascular disease (CVD) is essential for health management and policymaking. In this paper, we used a regression model to estimate the future impact of demographic changes on the burden of CVD in Iran during the next two decades.

**Methods:**

Disability-adjusted life years (DALY) were used to estimate the future burden of CVD in Iran. A regression model was used to estimate DALY caused by CVD in the Iranian population aged 30–100 yr, stratified by age group and sex. The predicted population of Iranians aged ≥ 30 yr was entered into the model and DALY were calculated over 2005–2025. To assess the areas of uncertainty in the model, we did sensitivity analysis and Monte Carlo Simulation.

**Results:**

In the year 2005, there were 847309 DALYs caused by CVD in Iranian adults aged ≥ 30 yr. This figure will nearly be 1728836 DALYs in 2025. In other words, just because of the aging, DALY related to CVD will increase more than two-fold in 2025 compared with 2005. The burden of CVD was higher in men (443235) than in women (404235) in 2005; but in 2025, the difference will be less (867639 vs. 861319).

**Conclusion:**

The burden of CVD will increase steeply in Iran over 2005–2025, mainly because of the aging population. Therefore, more attention is needed to deal with the impact of CVD in the following decades in Iran.

## Introduction

Gathering information about various causes of death is a valid tool for monitoring health promotion and defining the priorities of community health. Furthermore, a reliable and comparative explanation of the burden of diseases has an important role in decision making and planning in health systems (1, 2).

Non-communicable diseases were responsible for 60% of all deaths in 2008 (3). Approximately 80% of these deaths occurred in developing countries (4–7). Cardiovascular disease (CVD) is one of the chronic, non-communicable diseases responsible for more than 12% of the global disease burden (8). The number of people suffering from CVD is on the rise over the coming years (9–11). This increasing trend is not limited to low and middle-income countries. Even in high-income countries, the leading cause of disease burden is attributed to CVD (6, 11). In most high-income countries despite the increase in the burden of CVD, age-adjusted mortality rates of CVD have declined progressively during the recent decades (12, 13). The main reason for this reduction is related to both primary and secondary preventive interventions and ongoing health promotion programs (6, 14). In these countries, the combination of two factors: the aging population and development of medical technology have led to increased health care costs (15). Therefore, in high-income countries, CVD is considered as a top priority disease to be managed (16). However, in middle- and low-income countries, CVD is the main cause of death and the most important source of disease burden, neglected.

Compared with developed countries where 80% of deaths occur in retirement ages, in developing countries, most deaths occur in active ages (8, 11, 17–20). Premature death and loss of active years of life not only caused a great financial burden for affected individuals and their families but also for the society as a whole (19).

Despite all the above-mentioned consequences of CVD, it is one of the most preventable diseases as over 80% of early CVD are avoidable (20–22). Iran (developing country) has experienced major demographic changes in recent decades. It will have an increasing and aging population in future decades (23, 24). In fact, because of epidemiological and demographic transitions, patterns of morbidity and mortality have changed in this country. The first National Burden of Disease (NBD) study in Iran showed that 58% of DALYs were owing to non-communicable diseases in 2003 (25). Currently, CVD is the leading cause of death in Iran (25–28). Furthermore, 50% of all deaths per year and 79% of deaths related to chronic diseases are attributed to CVD (26, 29). Unfortunately, in Iran similar to other low and middle-income countries, a large proportion of premature deaths related to CVD occur during productive age.

Because of increased prevalence of cardiovascular risk factors such as inappropriate diet, insufficient physical activity, and smoking (30), as well as demographic changes and nature of age-related CVD, that will be one of the leading health-related problems in Iran.

Having an outlook about the disease profile facilitates the prioritisation in health system. Insight into the future structure of population and burden of CVDs result in making a reasonable and practicable decision by stakeholders of health system (31). To make the best decision and intervention program to reduce the burden of CVD, it is necessary to have complete information about the status of the disease and recognize the factors affecting the disease burden (15). Predictive studies have provided access to such information. Futures studies such as modeling have gained growing acceptance in recent years, partially in the area of health policy. Modeling studies have been extensively used in order to discover the potential importance of factors with deficient or absent data (32). Since low and middle-income countries have limited resources; financial, time, and human 7 such approaches (future studies) are highly important in resource allocation, management, and prioritization in such countries (33).

In the present study, we used a regression model to estimate the future impact of demographic changes alone on the burden of CVDs in Iran during 2005–2025. In this study, levels of CVD risk factors excluding age were held constant during the study period. All estimates were obtained according to uncertainties in population projections and numbers of deaths caused by CVD (conducted sensitivity analysis).

## Methods

The method of this study consisted of two main processes: Estimation of Iran’s population by age and sex based on demographic assumptions, and estimation of the burden of CVDs during 2005– 2025.

Briefly, we predicted the population of Iran up to 2025, then we applied the age and sex specific disability-adjusted life years (DALY), extracted from the national study in 2003, on the predicted age and sex distributions for the following years to estimate the overall burden in the whole adult population. Data extracting and data analysis was performed in 2016.

### Population Estimations

As there was no estimate of Iranian population aged over 30 yr during the coming years, the population projection of the 2015 revision of World Population Prospects (United Nations, 2015)” was used to predict the Iranian population by age and sex during 2005–2025. The Prospects has provided all demographic predictions for low, medium, constant, high, and instant replacement fertility scenarios (34).

In the instant replacement scenario, total fertility in Iran would be at the replacement level for each five-year projection period. In the high fertility scenario, Iran would have no fertility reduction or only an incipient decline until 2010. In the medium fertility scenario, total fertility in Iran would decline but it is still above 2.1 children per woman during 2005–2010. In the low fertility scenario, total fertility in Iran would be equal to or below 2.1 and finally, the constant scenario means that total fertility remains constant at the estimated level for the period of 2005–2010. Thus, the expected total fertility rates in Iran considering the mentioned demographic assumptions will be 2.1, 1.86, 1.36, 0.86, and 1.77 in 2025, respectively (Table 1).

**Table 1:** Fertility scenarios to predict population (World Population Prospects: the 2015 revision)

**Time period**	**Medium**	**High (optimistic)**	**Low (pessimistic)**	**Constant**	**Instant replacement**
2000–2005	1.96	1.96	1.96	1.96	2.1
2005–2010	1.77	1.77	1.77	1.77	2.1
2010–2015	1.59	1.84	1.34	1.77	2.1
2015–2020	1.45	1.85	1.05	1.77	2.1
2020–2025	1.36	1.86	0.86	1.77	2.1

The population estimates were obtained using Spectrum software, version 3.14 organized by the USAID/Health Policy project through source of statistics coming from projections of the “United Nations (23). Then we derived the population predictions by sex and age groups considering the four demographic assumptions within 5-yr intervals.

To ensure the accuracy of the population estimates, the estimated population of national census database for the base year (2005) was compared with the population estimates from the latest census conducted in 2005. The results revealed the similarities of the two estimates.

### Estimation the burden of cardiovascular disease

DALYs was used to estimate the future burden of CVD in Iran. Based on a systematic literature review, a few studies had been done to estimate the burden of diseases in Iran (25, 35–36). Only one of the studies retrieved was useable in our project, the study of disease burden in Iranian population was conducted by the Iranian Ministry of Health and Medical Education in 2007. This study was done for the population of 2003. In order to estimate the DALYs caused by CVD on the Iranian aged 30–100 yr, stratified by age and sex, we used a regression model.

Because DALYs caused by CVD that existed in age groups ranged up to 10 yr (34–44, 45–59, 60–69, 70–79 and >80), to estimate the burden of CVD in age groups with the 5 intervals, by using a regression model. First, the mean age of each age groups with large intervals was calculated, CVD DALYs related to each of the age groups extracted, slope and constant coefficients and then linear regression equations related to these variables (DALYs & Age) were calculated. All these steps were accomplished for both sexes and separately for males and females. In this way, three equations were obtained: an equation for estimating the total burden of CVD in Iranians aged over 30 yr

$$\left(\mathrm{DAL}Y_{\mathrm{bothsex}}=-5047.25+173.71*\;\mathrm{Agegroup}\right)$$

an equation related to burden of disease for all men over 30 yr

$$\left(\mathrm{DAL}Y_{\mathrm{male}}=-4896.54+171.17*\;\mathrm{Agegroup}\right)$$

and finally, the last equation was related to burden of disease for women over 30 yr

$$\left(\mathrm{DAL}Y_{\mathrm{female}}=-5190.07+176.11*\;\mathrm{Agegroup}\right)$$

After attaining the appropriate equations, the burden of CVD for every 5-yr age group in the population of 2003 estimated using interpolation method. At last, the predicted population of Iranians aged ≥ 30 yr was entered into the model, and DALYs attributed to CVD at baseline (2005) and the following 20 yr were predicted using direct age standardization method. Then, age and sex adjusted burden of CVDs for people aged ≥30 yr from 2005–2025 was calculated.

In order to evaluate the net effect of changes in population structure, we assumed that the distribution of age and sex-related risk factors (including direct and indirect variables/factors) were set constant at the baseline.

### Statistical analysis

Data analyses were performed using Stata software version 11.2 (Stata Corp, College Station, TX, USA) and MS Excel (2007).

### Model validation

Output of modeling studies depends on many factors and valid estimations are hardly attainable. To overcome this issue and achieve optimal predictions, traditional Sensitivity analyses and Monte Carlo simulation were applied to examine uncertainty regarding population predictions and number of deaths related to CVD.

### Sensitivity analysis

To address the uncertainty in the population estimates, Sensitivity analysis was done across two values for population estimates; 2% and 5% error. Reviews undercounting rates taken into account for data on CVD death were as follows: 5%, 10% and 20% (37, 38). The years of life lost because of CVD in the period 2005–2025 were calculated considering the six scenarios. In the first scenario, we assumed that population estimates had 2% error and mortality rates calculated in 2003 had 5% undercounting. The assumptions of the second scenario were probability of 2% error in population estimates and 10% under-counting in mortality rates calculated in 2003. In the third scenario, error in population estimates was assumed 2% and maximum undercounting in death rates were 20%. Other scenarios tested in this study included: Probability of 5% error in population estimates and 5% undercounting in mortality rates calculated in 2003, probability of 5% error in population estimates and 10% undercounting in mortality rates calculated in 2003, and probability of 5% error in population estimates and 20% undercounting in mortality rates calculated in 2003.

Values of the parameter (DALYs) estimated that by five interval age groups than 95% confidence intervals for each of these values were calculated using Monte Carlo simulation. This project was a type of secondary study (Modelling study), so it did not require ethics committee approval.

## Results

### Changes of population pyramid

**Fig. 1:**
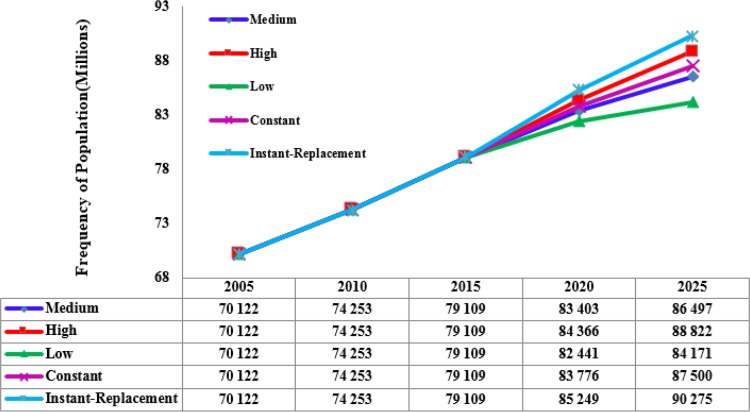
Prediction of Iranian population under 5demographic assumptions (2005–2025) based on population predictions of UN

We found that considering any of the four demographic assumptions, Iran’s populations would be growing over the coming years. In 2005, the total population of Iran was 70, 122, 200 while the estimated population in 2025 under low, high and instant replacement variant assumptions would be 84171000, 88822000, and 90275000 (Fig. 1).

**Fig. 2:**
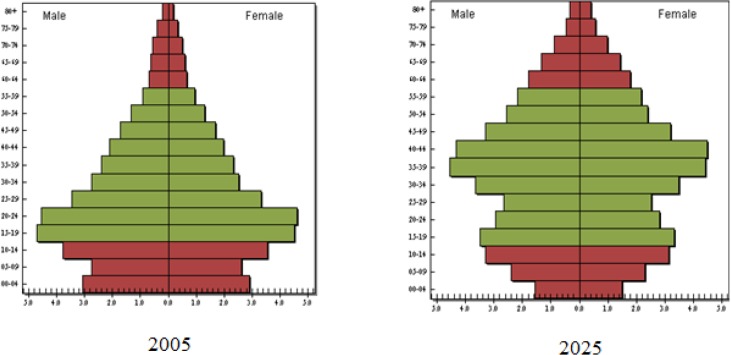
Age and Sex pyramid in 2005 compared with 2025

In 2005, the largest proportion of the population belonged to the 15–29 yr age group while it would shift to the age group of 30–44 yr in 2025 (Fig. 2).

Iran will face to an overall population increase and aging of adult population in the future decade. At baseline (2005) the proportions of people ≥30 yr were 38.01% of all Iranian population, while in 2025 the total adults aged ≥30 yr will be 59.12% of all population. The population over 65 will increase from 4.93% to 7.82%.

In 2005, the population size of men aged ≥30 yr compared with the age-matched female population was more (889000 people more). However, over time, the difference will be reduced and even reversed in 2025. In 2025, the estimated number of women aged ≥30 yr would be more than men do in the same age group (nearly 530000 people).

### Findings related to burden of CVD

In 2005, there were 847309 DALYs because of CVD for Iranian adults aged ≥30 yr. This figure will be about 1728836 DALYs in 2025 if the age-specific death rates between 2005 and 2025 remain constant at the level of the base year (2005).

**Fig. 3:**
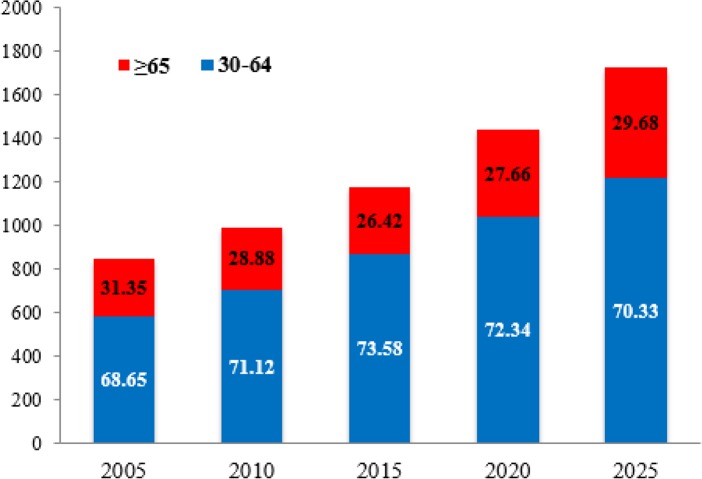
Predicted annual CVD burden by age 30–64 yr and ≥ 65 yr in iranian adults (2005–2025)

**Fig. 4:**
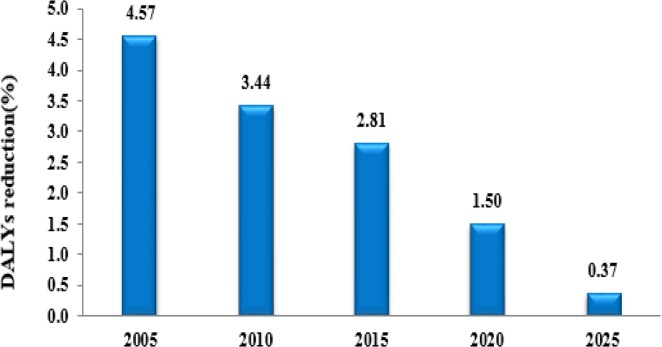
Estimated reduction in the years of life lost because of CVD in men compare with women in 2025. (Women as refrence group)

In other words, with the aging of the Iranian population, DALYs related to CVD will increase more than two-fold in 2025 compared with 2005. For 2025 the aging of the Iranian society will be reflected in estimated rise in CVD burden in the <65 yr age group (Fig. 3). In 2025 more than 70.3% of all burdens of cardiovascular disease would occur in the population aged <65 yr. The burden of CVD was higher for men compared with women in 2005 (443235 vs. 404539). In 2025, once again attributed burden of study diseases will be more in men than in women. Although this difference will be a significant reduction. In fact, years of life lost because of CVD in men will be 0.37 % (6320 DALYs) more than women in 2025 (Fig. 4).

**Fig. 5:**
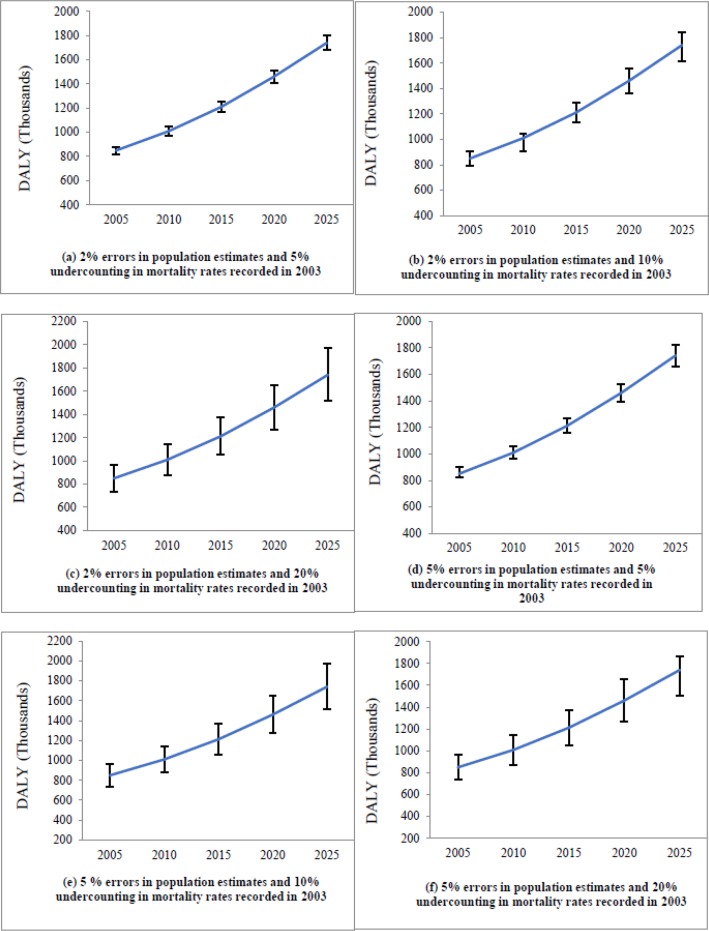
Point estimation and 95% confidence interval of years of life lost because of CVD between 2005–2025, considering 3 different errors in population estimates and 3 different undercounting in mortality rates recorded in 2003 (6 scenarios)

In general, years of life lost because of CVD in all age groups will have an increasing trend through 2005–2025. The most dramatic increase will be observed in age groups 55–64, 45–54, and 30–44-year-old, respectively. In the 65–74 year age group, a steep increase in DALYs would begin from 2015. The minimum increase will be in the 75–84 year age group (Table 2). Fig. 5 shows the point estimation and 95% confidence interval of the predicted years of life lost because of CVD from 2005 to 2025 in Iranian adults ≥30 yr old considering various scenarios discussed in the Methods section.

**Table 2:** Projections of disability adjusted life years between 2005 to 2025 by sex and small age groups

**Age group (yr)**	**2005**			**2025**		
	Both sexes	Male	Female	Both sexes	Male	Female
30–34	30850.99	17592.98	13433.99	41757.25	23527.20	18411.35
35–39	68507.62	37086.87	31600.86	132672.61	66592.20	66057.05
40–44	95558.91	50673.19	45005.53	208583.40	100450.50	108037.26
45–49	108733.84	56657.05	52138.80	212397.08	103886.60	108507.95
50–54	113088.04	57927.77	55185.06	199796.52	104880.63	94980.95
55–59	87322.57	43504.07	43822.10	211773.70	112366.88	99428.81
60–64	77652.77	39262.72	38387.47	208798.32	104925.07	103868.01
65–69	84032.47	44516.68	39494.76	188886.63	92310.51	96575.24
70–74	79688.68	42916.44	36736.49	156602.38	75747.64	80858.27
75–79	55083.56	29088.84	25969.64	85485.92	41134.46	44355.27
80–84	32265.20	16661.19	15591.39	46607.62	23828.10	22764.25
85–89	11560.08	5832.10	5724.76	25284.12	12953.68	12320.66
90–94	2501.39	1282.16	1218.16	8707.87	4334.81	4371.24
95–99	463.80	233.62	230.04	1483.32	700.86	782.72
100+	0.00	0.00	0.00	0.00	0.00	0.00
Total	847309.93	443235.68	404539.03	1728836.74	867639.14	861319.02

In the most optimistic scenario in which 2% errors in population estimates and 5% undercounting in mortality rates recorded in 2003, the minimum and maximum of all years of life lost because of CVD in 2025 is estimated at 1680210 and 1802482 DALYs, respectively. In other words, approximately 104% increase in disease burden will occur.

Based on the maximum error in parameters (5% chance of error in population estimates and 20% undercounting in the number of registered deaths in 2003), the minimum and maximum estimated DALYs attributed to CVD for 2025 will be 1509230 and 1977039 yr of life lost respectively, which shows more than 72% increase in CVD burden.

## Discussion

Similar to other modeling studies, predicted values in this study have been made based on certain assumptions [2]. Based on the study findings, population of Iran is on the rise and aging. We projected that years of life lost owing to CVD will increase dramatically in the coming years compare to base year (1728836 vs. 847309 DALYs), even with the assumption of constant levels of CVD risk factors, according to predicted population growth and aging of Iranian society. The independent effect or consequence of aging on increasing cardiovascular disease will be greater during the next years.

Population predicts showed that in 2005 the frequency of men ≥30 yr was more than women. While in 2025, number of women will be more than men. Predictions of DALYs related to CVD by sex showed that the burden of CVD was higher for men (443235) compared with women (404539) in 2005. However, in 2025 the difference in CVD burden in men and women will be less. This finding is expected because in most countries life expectancy in women is more than men, therefore, in upcoming years number of aging women will be higher than men in similar age groups.

According to our findings, if mortality rates attributed to CVD continue similar to the year 2005, CVD will be the main health problem in the country, although these diseases are the leading causes of death in Iran at present.

In developing countries, CVD mostly happens in adults <60 yr (14, 39), in low and middle-income countries people are mostly affected by CVD during the working age compared with developed countries where a large amount of CVD burden belong to retired individuals (6, 14, 19). Even more in most developed countries, mortality rates attributed to CVD had impressive decline (19, 40, 41) partially in younger age groups.

According to the uncertainty analysis, the maximum and minimum increase in burden of CVD in the studied period will be 72% and 104%, respectively. Our DALYs predictions were nearly close to reality value because 95% confidence intervals for the estimated values were not wide in all scenarios.

To provide a comprehensive perception of the burden of disease it is necessary to evaluate demographic characteristics and epidemiological factors separately. The global burden of disease project was an international effort to measure the burden of diseases and injuries (42). All DALYs predictions in our study were calculated with assuming constant trend in CVD death at the level of 2005. Furthermore, the prevalence of CVD risk factors observed in 2005 will continue at the same ranges during 2005–2025. In other words, we predict burden of cardiovascular disease only followed by increasing proportion of old people in Iran.

Having insight into the status and future trends in health-related indicators is very important for policy making especially in developing countries with limited resources (7), because it enables policymakers to set priorities in health care system (42). Therefore, population predictions are important tools that help stakeholders and policy-makers to assess and forecast future requirements in health policy making.

Non-communicable diseases such as cancers and CVDs are age- and sex-related diseases. On the other hand countries with high young population such as Iran will face demographic changes in coming years so that proportion of old people in the society will increase. Such changes will result in increase in the incidence, prevalence, and burden of age- and sex-related diseases such as CVD. Currently, CVD is the main cause of death in Iran (28, 43, 44) and like other developing countries, the incidence of cardiovascular diseases happens during active age.

Even in low fertility scenario, (total fertility in Iran would be equal to or below 2.1 children per woman in 2005–2010), burden of CVD will be increased and this increase is more evident in women (10).

DALYs estimated in our study might be greater in coming years because of the following reasons:

1. There is heterogeneity in demographic behavior in different regions of Iran.
2. Similar to other developing countries, there is an increasing trend in the prevalence of risk factors of heart diseases (45).
3. Programs and preventive interventions are not uniform in various parts of the country.
4. Finally, there is an inequality in access to health care in different regions of Iran.

### Study Limitations

Our study had some limitations. The predictions attained in our study were based on the only existing evidence on the burden of the disease in Iran. They might be affected by some errors; although we ran sensitivity analyses and Monte Carlo simulation to address the uncertainty related to population predictions and number of deaths related to CVD.

As the frequency of risk factors such as obesity, smoking, physical activity, and stress will change over time, future studies considering such factors are recommended. Because of resource constraints and restricted access to reliable and valid data on CVD morbidity and considering the cost effective nature of predictive studies, predictive and modeling studies are a priority (19, 46, 47).

## Conclusion

Because of growing and aging population, the burden of CVD will increase steeply in Iran during 2005–2025, even if the epidemiological factors do not decrease during that period. In addition, the highest disease burden will persist in the working age (<65 yr). Aging of population is an inevitable phenomenon; however, premature CVD can be prevented by focusing on coherent, organized health policies and planning such as primary intervention, screening, and early detection of high-risk groups.

## Ethical considerations

Ethical issues (Including plagiarism, informed consent, misconduct, data fabrication and/or falsification, double publication and/or submission, redundancy, etc.) have been completely observed by the authors.
